# Applications of Genomic Tools in Plant Breeding: Crop Biofortification

**DOI:** 10.3390/ijms23063086

**Published:** 2022-03-13

**Authors:** Inés Medina-Lozano, Aurora Díaz

**Affiliations:** 1 Departamento de Ciencia Vegetal, Centro de Investigación y Tecnología Agroalimentaria de Aragón (CITA), Universidad de Zaragoza, Avda. Montañana 930, 50059 Zaragoza, Spain; imedina@cita-aragon.es; 2 Instituto Agroalimentario de Aragón—IA2, Centro de Investigación y Tecnología Agroalimentaria de Aragón (CITA), Universidad de Zaragoza, 50013 Zaragoza, Spain

**Keywords:** biofortification, breeding, crop, cisgenesis, intragenesis, metabolic GWAS (mGWAS), single-nucleotide polymorphisms (SNPs), trangenesis

## Abstract

Crop breeding has mainly been focused on increasing productivity, either directly or by decreasing the losses caused by biotic and abiotic stresses (that is, incorporating resistance to diseases and enhancing tolerance to adverse conditions, respectively). Quite the opposite, little attention has been paid to improve the nutritional value of crops. It has not been until recently that crop biofortification has become an objective within breeding programs, through either conventional methods or genetic engineering. There are many steps along this long path, from the initial evaluation of germplasm for the content of nutrients and health-promoting compounds to the development of biofortified varieties, with the available and future genomic tools assisting scientists and breeders in reaching their objectives as well as speeding up the process. This review offers a compendium of the genomic technologies used to explore and create biodiversity, to associate the traits of interest to the genome, and to transfer the genomic regions responsible for the desirable characteristics into potential new varieties. Finally, a glimpse of future perspectives and challenges in this emerging area is offered by taking the present scenario and the slow progress of the regulatory framework as the starting point.

## 1. Introduction

Malnutrition is known to be a global public health problem and it has worsened with the COVID-19 pandemic. In 2020, about 768 million people in the world faced hunger, around 118 million more than in 2019 [1]. In addition, around 2.37 billion people (nearly one in three people in the world) suffered food insecurity (i.e., an inadequate access to safe, nutritious and sufficient food) in 2020, almost 320 million people more in just one year [1]. In fact, it is the first time that food insecurity has increased in North America and Europe since 2014 [1]. However, malnutrition is not only caused by the lack of food but also by a low dietary intake of essential nutrients (micronutrients included), known as hidden hunger [2]. This problem affects mainly developing countries in which the diet is usually based on more affordable major staple crops, characterized by a low micronutrient content. That being true, malnutrition is also present in developed countries, although in this case it is possibly due to unhealthy habits, such as extreme weight loss diets or substance abuse. It does not alleviate this situation given the fact that crop breeding has been mainly focused on increasing production, incorporating resistance to diseases, and enhancing tolerance to abiotic stresses, which has resulted in commercial varieties with low nutritional value [3].

Biofortification, i.e., the development of food crops with a high nutritional value per se through both conventional breeding and modern biotechnology techniques, could help in preventing hidden hunger. Micronutrients, minerals [4,5,6,7,8,9,10,11,12], vitamins [13,14,15,16,17,18,19,20,21,22,23,24,25,26,27,28], or both [29,], are the most common nutritional targets for biofortification strategies, though the improvement in fatty acid composition [30,31,32,33] and the increase in essential amino acids [34,35,36,37] and antioxidants [38,39,40,41] have also been recently included as aims of biofortification programs. This strategy carries multiple advantages. For example, it is a cost-effective approach, as shown by studies that report that for every dollar invested in the development of biofortified crops, as much as USD 17 of benefits may be obtained [42]. This is because, after a one-time investment to obtain the biofortified crops, they are able to synthesize larger amounts of the particular compounds without the need of adding any external micronutrients (fertilizers), which was the case in classical fortification. Therefore, as well as economic benefits, biofortification also brings environmental benefits. Moreover, it seems that breeding for a higher content in micronutrients does not entail a yield penalty [43,44]. This could be really helpful in developing countries, especially in areas with a limited access to marketed crops, as farmers could grow biofortified crops in the same way as conventional crops. Consequently, biofortification could be considered a sustainable and long-term solution to hidden hunger. In fact, the expected increase in population up to 9.7 billion by 2050 [45] makes it even more necessary.

Nevertheless, since the biofortification of a crop is tackled until the product is released to the market, a series of key steps have to be taken. The first would be to choose the species and the micronutrient to be enhanced. To maximize the positive impact on society, most consumed crops should be the target. This is what has been actually happening as, among the biofortified crops already developed, we can find staple crops, such as cereals (barley, maize, rice, and wheat) and beans, and some of the most consumed vegetables (tomato and potato) and fruits (apple and banana). One of the first steps consists of an evaluation of germplasm for their content in nutrients and health-promoting compounds; thus, outstanding alleles for those metabolic traits can be selected. Alternatively, the variability can be generated through induced mutagenesis (widely used in plant breeding since optimized during the second half of the 20th century) or by other more modern techniques of gene editing (i.e., clustered regularly interspaced short palindromic repeats (CRISPR)-associated system (CRISPR/Cas)). Secondly, genetic studies are usually conducted and molecular markers have to be developed to associate the trait of interest to the genomic regions. Finally, the allelic variants responsible for an increased content of the particular phytochemical have to be introduced to obtain the biofortified crop, either by conventional breeding or by modern biotechnology techniques. In this review, we will describe these steps in depth and, within the modern methods to introduce the allelic variants responsible for the increase in the specific compound, we will focus on transgenesis, cisgenesis, and intragenesis. Other simultaneous efforts will have to be made in order to ensure success both in the commercialization of the biofortified product and in the impact on consumers’ health. For the first goal, studies of market potential and consumers’ behavior and acceptability will have to be undertaken in advance, as was the case of selenium-biofortified apples [46] and iodine-biofortified fruits and vegetables [47], for example, both in Germany. This point is especially important in the case of controversial goods, such as transgenic biofortified food. That should be accompanied by promotion campaigns to make the product’s beneficial properties public, as the one carried out with the orange-flesh sweet potato biofortified in pro-vitamin A in Ghana and Nigeria [48]. For the second objective, analyses of micronutrient bioavailability and their efficacy of conversion in the human body will have to be performed, as reported in intervention studies which supply vitamin A-biofortified maize to Zambian children with promising results [49].

Taking all the above into account, the present review aims, firstly, to summarize the genomic tools available to explore the variability through single-nucleotide polymorphism (SNP) genotyping, and the analytical methods to determine the phytochemical profile and/or content of plant food. Secondly, a compendium of the researches carried out on the genomic association of metabolic data in crops is also presented here. Thirdly, different methods used to transfer the genomic regions responsible for a raise in the compound synthesis to the crops in order to create new biofortified varieties are shown, as well as some examples of their applications. These methods are either encompassed in conventional breeding strategies or modern biotechnology approaches, such as transgenesis, cisgenesis, and intragenesis. Finally, an overview of the current regulation and the future prospects of developing nutritionally enriched crops is also offered.

All the information needed to deal with the subjects mentioned above is obtained through searches in public databases and webpages, as described in Supplementary File S1.

## 2. Exploring Biodiversity: Searching for Outstanding Material

### 2.1. Genomic Diversity Enquired by SNP Genotyping

**Figure 1:**
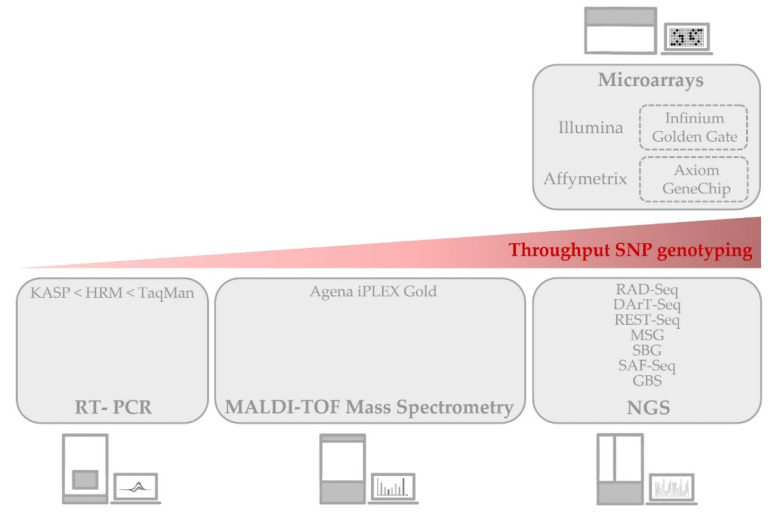
Comparison of the SNP genotyping techniques most commonly used in crops grouped by the platforms in the throughput level.

SNPs are not only the most frequent sequence variations among all practically genomes [50], but also the most amenable to automation. Even if a long list of molecular markers, and, more specifically, genetic markers, has been used in plant breeding since the 1980′s (restriction fragment length polymorphisms (RFLPs), random amplified polymorphic DNAs (RAPDs), amplified fragment length polymorphisms (AFLPs), simple sequence repeats (SSRs), inter-simple sequence repeats (ISSRs), cleaved amplified polymorphic sequences (CAPS), etc.) [51], all of them have been unarguably ousted by SNPs. Their predominance is also a consequence of the development of next-generation sequencing (NGS), including second- and third-generation sequencing (SGS and TGS), mainly SGS, which evolved from the sequencing of short DNA fragments (first-generation sequencing, FGS) to high-throughput technologies (SGS) and, finally, single-molecule sequencing (TGS). This soon made necessary high-throughput SNP genotyping platforms that could produce a massive volume of data more cost-effectively in a short period of time. Among the wide variety of techniques developed to genotype SNPs and the different detection methods coupled to them, we will highlight those more commonly used nowadays with crops and those that process a medium (normally, in the laboratory) to high number of markers and samples (commercial platforms). All of them are based on hybridization, amplification, sequencing, or a combination of them, and they have been grouped according to the type of platform employed (Figure 1).

#### 2.1.1. SNP Genotyping Microarrays

Among the assays available, the Affymetrix (Axiom) is a hybridization-based microarray that uses probes for both alleles. Independent of the allele at the particular locus, both probes hybridize with the DNA sample though the signal become dimmer in the case of a mismatch. So, the genotype of each SNP marker is called by the probes, showing the highest intensity in their signal. SNP Affymetrix arrays (either Axiom or GeneChip) have been used in a number of food crops, including cereals (maize [52,53], rice [54,55], rye [56], and wheat [57,58]), horticultural crops (chickpea [59], lettuce [60], potato [61], soybean [62], and strawberry [63]), and woody crops (apple tree [64] and peanut tree [65]), among others.

In the Illumina BeadArray (Infinium), the silica beads are coated with probes targeting a specific SNP locus. They bind the region just upstream the polymorphic site. Then, by single-base extension (SBE), a labelled nucleotide will be incorporated, emitting a different signal depending on the base. Illumina developed other BeadArray (GoldenGate) that uses fluorescent universal primers that hybridize to the allele-specific oligos. These technologies have been extensively used to discover and genotype SNPs in food crops, including cereals (barley [66], maize [67,68], oat [69], rice [70,71], and wheat [72]), oil crops (oilseed rape [73] and sunflower [74,75]), horticultural crops (cowpea [76], potato [77], tomato [78], and soybean [79]), and woody crops (apple tree [80,81], cherry tee [82], peach tree [83,84,85], pear tree [86], and vine [87,88]), among others.

The immobilization of samples, probes, ddNTP, etc. on chips (depending on the technique) is what makes interrogating hundreds of thousands or even millions of markers simultaneously feasible (Figure 1). In both cases, there are predesigned chips for some crops, which is the most affordable choice, but there is also the possibility of designing custom chips with the SNP markers of interest.

#### 2.1.2. Real-Time PCR for SNP Genotyping

One of the commercially available assays within this category is the TaqMan SNP genotyping. This technology is also based in DNA hybridization and amplification, the signal is generated by fluorescence resonance energy transfer (FRET), and it is amenable to automation by real-time PCR though it does not reach the same high-throughput format than microarrays (Figure 1). Briefly, two allele-specific probes are designed for each SNP locus with two different fluorescent dyes attached to them. When the probe is free, the fluorescence is suppressed by quenching. Only when the probe perfectly hybridizes with the DNA fragment containing the SNP allele and is extended by PCR, the fluorophore is released by the exonuclease activity of the DNA polymerase and its signal is captured by the appropriate detector. These techniques have been mainly used in plants to diagnose pathogens and, to a smaller extent, to identify transgenes and detect food frauds, though there are also some cases where they are used to study the genetics behind some traits of interest in food crops, such as the presence of anthocyanins in potato skin [89].

As the previous one, the Kompetitive allele-specific PCR (KASP) is also a FRET method that makes use of hybridization and amplification though, unlike the TaqMan assay, the reagents for the allele-specific amplification, on the one hand, and the dye and quenchers, on the other, act in two phases. During a first round of PCR, the allele-specific and the common reverse primer amplifies the region by harboring the target SNP. After this, one of the fluor-labelled oligo that was quenched until now binds as a tail to the corresponding amplified allele, generating a fluorescent signal. KASP assays have been extensively used in different crops, mainly cereals, becoming very helpful for MAS in wheat [90,91,92,93,94,95,96,97,98,99], barley [100], rice [101,102,103,104], sorghum [105], pea [106], watermelon [107,108], faba bean [109], tomato [110,111], and *Brassica oleracea* (cabbage, broccoli, kohlrabi, and Chinese kale [112]).

Another methodology included here is the high-resolution melting (HRM) analysis. After the amplification by PCR of the region containing the SNP of interest in the presence of a dye that binds to double-stranded DNA, the products are melted into a single strand. This then causes the release of the dye and a decrease in its fluorescence. The real-time PCR is able to detect those changes and generate a melt curve that is different for each of the genotypes at the SNP locus. Apart from cultivar identification, species authentication and pathogen diagnose, HRM has also been used for MAS to enhance the quality of soybean [113], rice [114], strawberry [115], and barley [116].

These methods normally do not reach the same high-throughput format than microarrays (Figure 1). However, nowadays, there are TaqMan and KASP arrays which help to process a high sample throughput for mid-density genotyping. In the case of TaqMan SNP genotyping, there are pre-designed and custom assays. Regarding HRM, as of recent, there are no commercial panels; however, it is the user who is in charge of designing and carrying out the assays. In the case of HRM and TaqMan (but not KASP) analyses, a low degree of multiplexing is possible (i.e., duplex).

#### 2.1.3. Mass Spectrometry SNP Genotyping

Primers are designed immediately adjacent to the SNP locus and an SBE is carried out using mass-modified dideoxynucleotide terminators. The mass of the allele-specific product is determined by using matrix-assisted laser desorption–ionization time-of-flight (MALDI-TOF) mass spectrometry. Like all the other SNP genotyping technologies, this is used with identification purposes in crops. Besides, it is applied in MAS for quality traits in cereals, such as barley [117], rice [118], legumes (including pea) [119], and mung bean [120].

This is a high-throughput technology (Figure 1) which can process thousands of samples per day, which also allows the simultaneous amplification and detection of multiple markers per reaction (i.e., Agena iPLEX Gold, previously known as Sequenom iPLEX Gold). This method avoids the problems derived from a background signal typical from those based on hybridization. As the previous ones, this type of assay can be custom-designed.

#### 2.1.4. SNP Analysis by NGS

With the increasing affordability of sequencing methods, these SNP genotyping platforms based on sequencing are becoming very popular. The main strategy nowadays consist of building reduced representation libraries (RRLs). By reducing the complexity of the targeted genome (normally digesting it with restriction enzymes), the depth of the sequencing can be increased. Among all the developed methods, including restriction site-associated DNA sequencing (RAD-Seq), diversity array technology sequencing (DArT-Seq), restriction fragment sequencing (REST-Seq), multiplex shotgun genotyping (MSG), sequence-based genotyping (SBG), specific-locus amplified fragment sequencing (SAF-Seq), etc., one of the most widely used in crops is genotyping by sequencing (GBS). Briefly, the whole genome is fragmented using restriction enzymes and short-read sequencing is performed on the ends (paired-end sequencing). Libraries for each sample are prepared using different barcodes; thus, a multiplex approach in which thousands of genotype SNPs across thousands of samples simultaneously was possible (Figure 1). GBS is used in studies on some traits that influence the nutritional value of food crops, such as the soluble solid content in plum [121]; sugar and acid content in apple [122]; sugar and carotenoid content in melon [123]; and certain mineral content in maize [124], pea [125], and spinach [126].

As in the previous technologies, pre-designed assays are available for some crops though custom panels of markers are also possible.

Thanks to this profusion of technologies that are becoming more and more affordable, a large number of SNP databases in crops is made available (Table 1). The data that have been made public in this way feed back into the agrigenomic field, as they can be used by other researchers to design their assays. Some of them only include marker information, but others also supplied the genotypes in different accessions (cultivars and wild crops relatives) as well as other useful tools, including genetic maps, genome sequences, etc. Table 1 clearly shows a higher representation of staple crops (i.e., cereals), given the very intense genetic breeding in recent decades, though other crops with a great economic importance, such as fruit trees (i.e., within Rosaceae family) or vegetables (i.e., tomato), are also present.

For the above, SNPs are the preferred markers to both, carry out genetic studies and undertake breeding programs in crops. Actually, genotyping assays have been developed for a large number of plants, including all major crops.

### 2.2. Nutritional and Phytochemical Profiles Assessed by Analytical Methods

The “omic” era has also reached the characterization of food plants in terms of their nutritional content, making use of metabolomic technologies. Thus, it is now possible (though still prohibitive, in many cases) to obtain the complete profiles of phytochemicals in complex extracts in a high number of samples. In this way, the compounds are identified by metabolic profiling and then quantified by target analysis. This has huge potential in plant breeding, especially in crop biofortification, which is still to be fully exploited. The different techniques normally used for metabolome analysis are enlisted here very briefly, as that is not the main scope of this review.

#### 2.2.1. Mass Spectrometry (MS)

This is a very sensitive analytical technique, either used directly (non-hyphenated methods) or coupled with others (hyphenated methods), such as gas chromatography (GC), liquid chromatography (LC), or capillary electrophoresis (CE). In the first case, it is possible to process a high number of samples in a short period of time, though the identification capacity is limited. The hyphenated methods, on the other hand, are undoubtedly more powerful when it comes to identifying and quantifying metabolites, and there is also the possibility of reducing the running times by using more advanced techniques in chromatography (i.e., ultra-high-performance liquid chromatography (UPLC) instead of high-performance liquid chromatography (HPLC) [129]). In any case, the metabolite identification generally requires the availability of libraries in order to compare the spectra obtained.

#### 2.2.2. Nuclear Magnetic Resonance (NMR)

This is a very reproducible spectroscopic technique used to quantify metabolite levels. It allows a high-throughput process of samples, though it is generally less sensitive and has less resolution power than MS. Moreover, it is a non-destructive method, which makes it the perfect choice for studying the metabolome evolution (for instance, in different plant stages), instead of simply obtaining a snapshot of the plants at a particular moment.

Both techniques can be actually combined, resulting in the detection of a higher number of metabolites.

Until recently, the most common nutritional studies in food crops have focused on the quantification of a discrete number of compounds with a high impact in their nutritional value (targeted metabolic studies), though some widely targeted metabolomics analyses are starting to be carried out even in minor crops [130]. The initial steps which deal with the germplasm evaluation for nutrients and health-promoting compounds are essential for harnessing the biodiversity harbored by cultivated varieties, but also by breeding material and crop wild relatives. Some examples of these characterization works can be found in all groups of food crops, cereals [131], fruits [132], legumes [133], and vegetables [134,135], among others. In this sense, a considerable number of researches has compared different plant material within the same crop (for instance, landraces vs. commercial varieties) in order to identify outstanding accessions for future breeding programs aimed at enhancing the content of nutritious and beneficial compounds (reviewed in [3]). Metabolomic offers the opportunity to study the huge range of metabolites present in a sample (untargeted metabolic studies) and not only some specific compounds.

Another metabolome approach, apart from profiling commented above, consists of performing metabolomic fingerprints, where compounds are not individually identified. However, the metabolite profiles are compared among samples, for instance, to study the plants at different developmental stages [136] or under several biotic [137] and abiotic [138] stresses. We will not go into depth in the latter, as it is not related to the subject of this review, though it is noteworthy to mention that some studies use a combination of both approaches, i.e., by carrying out metabolomic fingerprint experiments in which the compounds are actually identified [136].

## 3. Association between the Traits of Interest and the Genomic Regions: Fishing for Genes

On one hand, one of the most useful and exploited genetic tools in crop breeding has been the linkage maps. Large SNP genotyping arrays have been used to build high and ultra-high-density genetic maps that allow the efficient marker-assisted selection (MAS) of beneficial alleles for the traits of interest. Nowadays, there are consensus and saturated genetic maps (mainly built with SSR and SNP markers) in virtually all the important crops and, in many cases, they are used to localize quantitatively trait loci (QTL). This fine mapping (often together with the QTL analysis) has led to the identification and cloning of the underlying gene(s), mainly in cereals (i.e., barley, maize, rice, and wheat), but also in some legumes (i.e., soybean) and vegetables (i.e., tomato) [139], though there are few cases for traits related to their nutritional value. An emerging application involves integrating metabolic/metabolomic and quantitative data to render metabolic QTL (mQTL). Until now, a number of these studies have been carried out, mainly in cereals (wheat, barley, rice, and maize) but also in oilseed rape and tomato [140]. As a result, numerous mQTL have been identified in those crops and some of them have eventually led to the identification of putative candidate genes controlling metabolic traits [140].

On the other hand, in genomics (the field that concerns us in this review), the whole genome of an organism is studied. As could be expected, the development of NGS technologies has led to a real boost for its applications, such as genome-wide association studies (GWAS). With the SNP genotyping by NGS, it is possible and affordable to rapidly scan markers across the complete genome of many individuals to find variations associated with a particular trait. In fact, the genotypes for thousands of SNPs are currently available for many crop species, as shown in Table 1. In order to make the most of all this already existing information, it can be combined with the results derived from the technology to analyze metabolites. In this line, researches which combine metabolic/metabolome and genome association results (metabolic/metabolomic GWAS, mGWAS) are starting to be carried out in crops (Table 2) and they are expected to become very helpful in genomic-assisted breeding programs by whole-genome selection and eventually in identifying some of the genes potentially influencing the nutritional value and the content of health-promoting compounds.

A potential drawback of this methodology, especially in the case of complex traits (as is the case of metabolism-related traits), is that the most significant variant obtained (i.e., allele of a SNP) is sometimes not responsible for metabolic differences. Actually, it is also common, as in any statistical analysis, to obtain spurious associations, for instance, when the trait heritability is low (high environmental effect). For this reason, it will still be necessary to carry out the validation of the candidate genes identified. In this sense, in many of those mGWAS involving compounds with a potential use to biofortify the respective crop (Table 2), other “omics” technologies, mainly transcriptomics, have assisted researchers in untangling the relationships between genotype and phenotype and in pinpointing the causal gene(s). Furthermore, it is also common to validate those findings by using mutants (knockout and/or overexpressing lines) and transgenic plants. Such an encompassing approach will undoubtedly speed up the process of obtaining healthier and nutritionally richer crops. Even if it is not the purpose of many of those studies, aimed at evaluating the metabolic changes that plants undergo during their development or to face environmental challenges (i.e., biotic and abiotic stresses), that knowledge about the genes responsible for the changes in metabolite contents is applicable in order to enhance the food in phytochemicals with beneficial properties.

## 4. Introducing Allelic Variants to Biofortify Crops

The last stages of the biofortification process in crop plants can be tackled through different approaches, including both conventional and modern biotechnology techniques, such as transgenesis, cisgenesis, intragenesis, or gene editing (i.e., CRISPR/Cas), in order to introduce genetic variation into the gene pool of the crop. Here, we will describe conventional breeding, transgenesis, cisgenesis, and intragenesis, as well as their applications in crop biofortification.

### 4.1. Conventional Breeding Assisted by Genomic Tools

**Figure 2:**
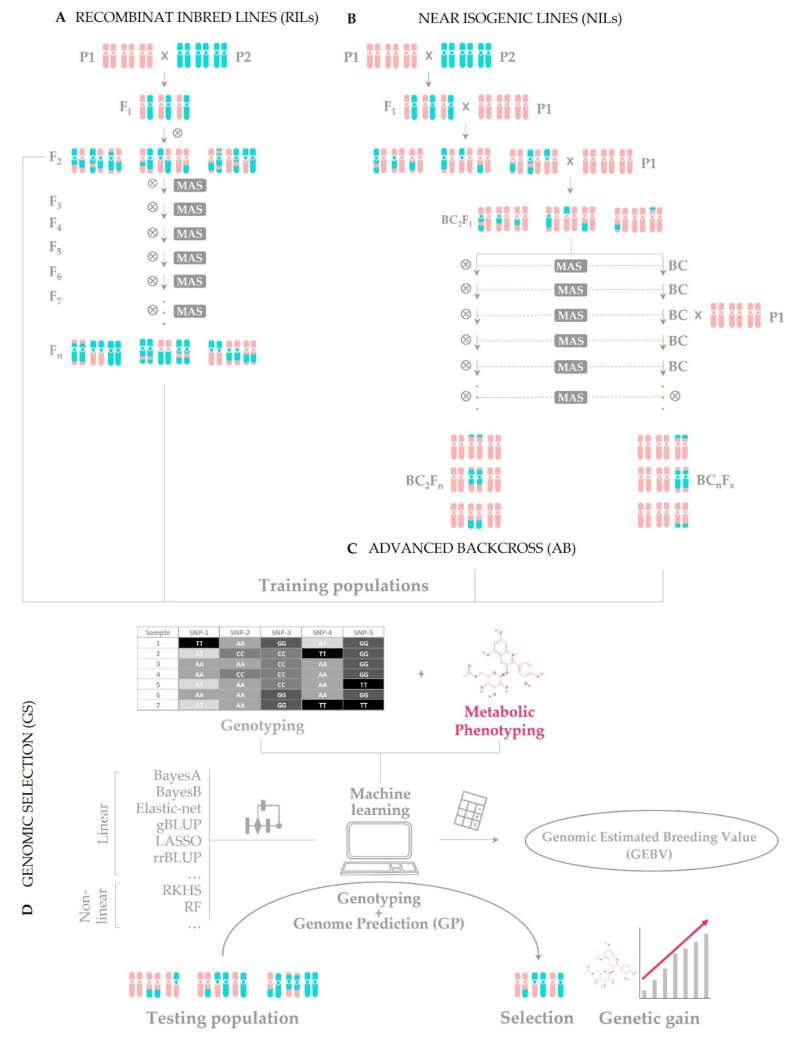
Construction of breeding populations: (**A**) recombinant inbred lines (RILs); (**B**) near isogenic lines (NILs); (**C**) advanced backcross; and (**D**) their use for Genomic Selection (GS). Only some of the possible crossing designs are shown.

Biofortification through conventional breeding is based on crosses within a sexually compatible group, specifically between donor plants with nutritional properties of interest and recipient ones with good agronomic characteristics. Many types of populations have been developed to perform genetic mapping, QTL identification, and association studies (i.e., both temporal (F_2_, backcrosses (BCs) and advance backcrosses (ABs)) and immortal (double haploid lines (DHLs), recombinant inbred lines (RILs), near isogenic lines (NILs), multi-parent advanced generation inter-cross (MAGIC), and nested association mapping (NAM)) ones). Among them, the most widely used in plant breeding to introgress DNA regions that harbor beneficial alleles for the trait of interest from the donor into the recipient parent are ABs, NILs, and RILs (Figure 2).

Several generations are needed, so it takes a substantial amount of time to obtain crops with the desired nutritional and agronomic characteristics by using this strategy. An emerging alternative approach to save time, effort, and money consists of carrying out a genomic selection (GS), as coined by Meuwissen et al. [183], based on a genomic prediction (GP) (Figure 1D). Instead of phenotyping at every stage of the population building (like in the MAS strategy), it is only carried out in what is known as training population (DHs, F_2_, marker-assisted recurrent selections, etc.). These data, together with genome-wide genotypic data from that same training population, are used to calculate the genomic estimated breeding value (GEBV) through processes of machine learning by means of different regression models. So, GEBV is a parameter used to quantify the genetic merit of a certain individual in order to improve the crop in the trait of interest. Finally, the GP is carried out with the data coming from genotyping the testing population (the breeding population) without the need to phenotype it. In this way, the individuals selected by the testing population are expected to show a genetic gain, i.e., an increase in performance thanks to the gene variant(s) responsible for the aforementioned trait. With this method, all markers are taken into account, not only those which show a significant association with the trait (as in MAS); thus, loci with little additive effects can also be detected. Until now, this approach has been scarcely used in crops for metabolite and nutritional content, such as in tomato [184] and wheat [185].

Furthermore, the chances of achieving biofortification by conventional breeding depends on the crop itself, since the strategy relies on the genetic variability available within its gene pool, which is usually limited in commercial varieties. This could be overcome by crossing plants with landraces or with more distant wild relatives that normally harbor higher genetic variability and, sometimes, can be richer in nutrients [3,186]. However, in some cases, it would be impracticable to obtain biofortified crops using conventional breeding. That would be the case when the genetic variability needed for a specific trait is insufficient within the gene pool, or when the investment of time and resources would be excessive, especially with non-diploid species, when the trait heritability is low or when linkage drag is unavoidable.

In spite of its limitations, conventional breeding is currently the most accepted method, as it is sustainable and it is not subject to regulatory obstacles. Nowadays, an important number of crops have been conventionally bred to enhance their nutritional content. In fact, several international organizations have initiated different programs to accomplish this objective. Harvest Plus, launched in 2003, is the most important one and is focused on enhancing the content of provitamin A, iron, and zinc in staple food crops across Asia and Africa [187]. It has managed to biofortify a large number of crops, many of which have been already released. Until 2019, there is a total of 242 across 30 developing countries [188]. Different studies have demonstrated the efficacy of biofortification through conventional methods, specifically increasing the content of micronutrients [189,190]. Furthermore, other smaller institutions are working on developing conventionally biofortified crops. For example, the International Potato Centre (CIP) has obtained, tested, and advertised an orange sweet potato enriched in provitamin A [191], and the International Maize and Wheat Improvement Centre (CIMMYT) has released different hybrid varieties with increased levels of the amino acids lysine and tryptophan through the incorporation of the naturally occurring mutation *opaque-2* (*o2*) into different maize varieties [192].

The assistance of genomic tools has facilitated the development of many conventional biofortified crops, as they allow breeders to exploit the available genetic variability more efficiently; thus, time and costs can be significantly reduced. Plant breeding has existed since plant domestication started around 10,000 years ago, and the selection carried out at the beginning merely attends to the phenotype. However, with the application of genetic and genomic tools, genetic variants can be associated with differences in phenotypes, which then enables the selection at early stages of the plant. For that, the construction of genetic maps has been essential, as previously mentioned. Many studies have found markers linked to genes or QTL which can control the content of nutritional compounds, for example, those related with carotenoid variation in sorghum [193], mineral micronutrients in beans and wheat [194,195], vitamins levels in different cereal crops [196], etc. Thus, individuals with the best gene combination have been identified and used as potential donors in breeding programs to enhance the content in micronutrients (minerals and vitamins) and health-promoting compounds (polyphenols, carotenoids) in all kinds of crops, including cereals, fruits, legumes, and vegetables (Table 3).

### 4.2. Modern Biotechnology Techniques

#### 4.2.1. Transgenesis

**Figure 3:**
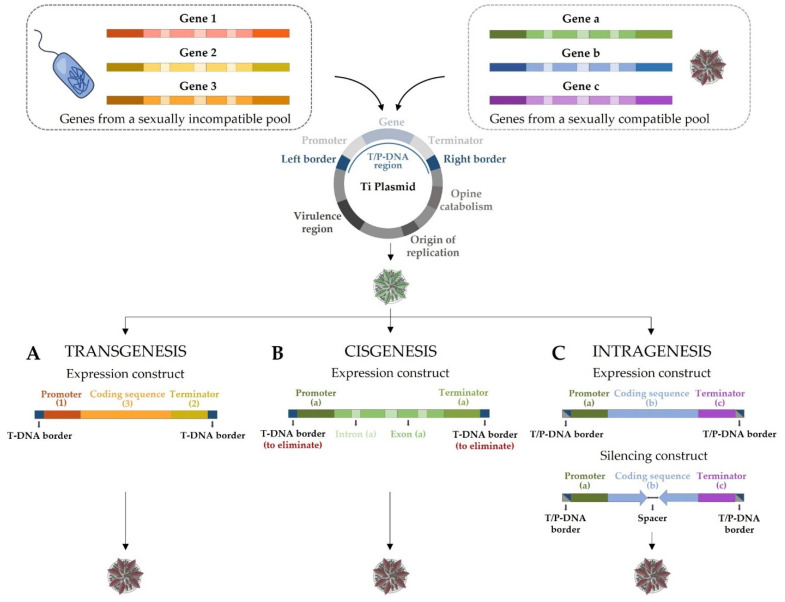
Schematic representation of three modern biotechnology techniques to introduce allelic variants of interest in a recipient organism: (**A**) transgeneis; (**B**) cisgenesis; and (**C**) intragenesis.

In biofortification, transgenic approaches consist of the transference of one or more alleles from genes responsible for the increase in the nutritional value from one or more organisms to the crop of interest. They are really helpful in overcoming the main handicap of conventional breeding, i.e., the limited genetic variation within the same or sexually compatible species [207]. Moreover, genetic transformation through transgenesis can achieve the expression of a gene independently of its origin, in terms of evolution, taxonomy, and even kingdom [19,208,209]. Hence, when a specific nutrient or a bioactive compound is not naturally synthesized in a crop, transgenesis is the only way to engineer the crop to produce it. Therefore, this strategy helps to exploit a much larger gene pool and transfers more than one gene and their regulatory regions simultaneously (Figure 3A). In this way, the crop can be enriched in more than one nutrient at the same time, as it has already been successfully engineered in rice [29]. However, it is important to take into account that some crops are recalcitrant to transformation and/or regeneration, for example, some cereals [210] or legumes [211].

Transgenic approaches require a lot of time and resources. The identification and characterization of the gene(s) are needed to eventually introduce them in the crop. Nevertheless, transgenesis is less time-consuming than the conventional alternative and more cost-effective than the agronomic fortification, which is ineffective in the long term because it requires regular applications of fertilizers [212]. This, together with the absence of taxonomic constrictions and the possibility of designing almost any synthetic gene, has resulted in a big number of biofortified crops developed through transgenic strategies (Table 3). One of the most remarkable examples is Golden Rice, obtained to alleviate the vitamin A deficiency [17]. It was the first application of transgenic biofortification, in which a carotenoid-free rice endosperm was genetically engineered to produce β-carotene (provitamin A) by expressing the genes codifying for the phytoene synthase and the carotene desaturase [17]. In addition, a clinical trial in humans has demonstrated that Golden Rice could be an alternative source of vitamin A for adults [213]. As in the case of conventional breeding, many different strategies have been applied to almost any kind of crop, including cereals, legumes, vegetables, fruits, and oilseeds, whereby the targets of biofortification are fatty acids, essential amino acids, and antioxidants, among others (Table 3).

The main disadvantage of these crops is the strict regulation to which they are subject to, at least, in Europe (more deeply described further on). However, some biofortified crops have gone beyond this limitation and they have been released. Some of these crops are cassava with improved levels of zinc, iron, β-carotene, or proteins, released by Biocassava Plus; canola with a higher availability of phosphate due to phytate degradation, released by BASF; and linseed enhanced in essential amino acids, released by the University Saskatchewan (Saskatoon, Canada).

#### 4.2.2. Cisgenesis and Intragenesis

Cisgenesis and intragenesis are approaches that, to some extent, were developed to overcome the main limitation of transgenesis—its strict regulation [214]. The gene pool exploited here can only come from naturally crossable species; therefore, they might be a suitable alternative to obtain biofortified crops.

On the one hand, the terms “cisgenic plant” were first introduced in 2006 as “a crop plant that has been genetically modified with one or more genes (containing introns and flanking regions such as native promoter and terminator regions in a sense orientation) isolated from a crossable donor plant” [215] (Figure 3B). This donor plant has to belong to the same species than the modified crop or to a sexually compatible species; thus, the gene pool available for cisgenesis is identical to the gene pool exploited by conventional breeding. Nevertheless, unlike conventional breeding, only the gene(s) of interest, and no undesired sequences (linkage drag), are transferred to the final cisgenic crops.

On the other hand, the terms “intragenic plant” were introduced in 2004 and they refer to the isolation of specific genetic elements from a plant, the recombination of these elements in vitro, and the insertion of the resulting expression cassettes into a sexually compatible plant [216] (Figure 3C). Intragenesis can also be carried out using constructs with RNA interference (RNAi) [28,206] or genes edited, for instance, by CRISPR/Cas, as this technology has been successfully used to edit the genome of crops [217,218]. Therefore, intragenesis provides the possibility of creating novel combinations that render higher variability and novel expression patterns to develop new genetically modified organisms (GMOs) with new properties that will not happen spontaneously in nature or through conventional breeding.

The main difference between cisgenesis and intragenesis is related to the regulatory regions. In cisgenesis, the transgene is a complete DNA copy of the gene as it can be found in the donor plant (with promoter, introns, and terminator) in the normal-sense orientation (Figure 3B). In intragenesis, there is not any requisite about these regulatory elements, as long as all the genetic elements come from crossable donor plants, so that they can be engineered before being used in the transformation (Figure 3C). Consequently, intragenesis is not considered as close to conventional breeding as cisgenesis.

In both cases, when *Agrobacterium*-mediated transformation is used, T-DNA borders (flanking sequences of the DNA to be transferred) can be also inserted in the plant genome. This is a controversial topic as some authors are in favor of using T-DNA borders, claiming that they are safe because they are short non-coding sequences that can be found in plant genomes naturally too [219]. The evident argument against T-DNA borders is that all DNA sequences integrated into the recipient plant should come from a sexually compatible DNA pool, as established by both cisgenesis and intragenesis definitions [215,216]. Thus, both cisgenic and intragenic crops should be free of those T-DNA borders, and also of selection markers and vector backbones, as both of them are supposed to be genetically modified plants that do not contain foreign genes (only genes coming from cross compatible species). Two alternative solutions have been proposed. First, plants without T-DNA borders can be selected just by carrying out a PCR. In fact, the integration rate of the T-DNA borders in the plant genome is relatively low, as is the case of transgenic potatoes carrying R genes for late blight, in which only 45% of transformants possessed T-DNA borders [220]. Second, T-DNA border-like sequences found in the plant genomes, known as P-DNA borders, can be used upstream and downstream the gene to be transferred [216,221]. A rearrangement of the original gene is thus required, as it was in the donor plant, which is why this option should only be chosen in the case of intragenic plants. Furthermore, the presence of T-DNA borders in both types of plants could be a problem for the public acceptance and in terms of regulation [222]. Regarding the other non-plant sequences, the use of selection markers is not necessary when the transformation efficiency is high [223] or the product codified by the introduced gene can be visually detected, including a pigmented compound (i.e., carotenes, anthocyanins) [224]. There are also methods to eliminate markers based on site-specific recombination (marker genes are flanked by specific recombination sites) [225], or by carrying out a co-transformation, which allows the segregation of the transgene and the marker gene in the progeny, as they are integrated in different positions of the genome [226].

In comparison to transgenesis, cisgenesis and intragenesis have two clear limitations (Table 4). The first one is that the available variability only exists in plants from the same sexual compatibility group, as in conventional breeding. However, this disadvantage could be overcome, to some extent, by gene edition (in the case of intragenesis) or by making use of the higher biodiversity present in landraces [3] or wild relatives [186]. The second limitation is the need to remove the selection markers and the vector backbones, which could be both time- and labor-consuming. On the other hand, although the three technologies are subject to the same regulation, cisgenic and intragenic crops are more accepted by the general public than transgenic ones [227,228,229].

When compared to conventional breeding, cisgenesis and intragenesis are considered fast alternatives to transfer genes between plants from the same sexual compatibility group, especially for species with long lifetimes and high heterozygosity levels (Table 4). Additionally, these two approaches are able to avoid linkage drag issues associated with backcrosses in conventional breeding, as only the sequences of interest are transferred (Table 4). Changes in the gene expression levels can also be achieved with both techniques (Table 4). The introduction of the complete natural gene (cisgenesis) and changes in promoters and terminators (intragenesis) may increase the levels of expression, whereas the use of silencing constructs (intragenesis) could reduce them. Moreover, new genetic variability can be generated with different combinations of genetic elements with intragenic approaches.

Although most of the new traits incorporated to relevant crops through cisgenesis and intragenesis are related to disease resistance [216,225] and abiotic stress tolerance [230], these strategies have been also applied with biofortification purposes (Table 3). For example, Holme et al. [202] obtained a cisgenic barley by inserting copies of a barley phytase gene (*HvPAPhy_a*). Those barley plants with a single copy of the gene showed a 2.8-fold increase in the phytase activity and an enhanced bioavailability of phosphate. A cisgenic potato was developed by suppressing the *starch phosphorylase L* gene through dsRNAi (double-strand RNA interference) technology to decrease starch degradation [203]. Then, the accumulation of reducing (glucose, fructose) and non-reducing (sucrose) sugars was lower in tubers stored at 4 °C. Finally, cisgenic red-fleshed apples, rich in anthocyanins, were developed by expressing the *MdMYB10* gene, a transcription factor involved in anthocyanin biosynthesis flanked by its native promoter and terminator [41]. In the case of intragenesis, potato is the most recurrently used crop for gene silencing strategies. In fact, the first intragenic application was the increase in amylopectin content in potato by silencing the *granule-bound starch synthase* gene (*GBSS*), responsible for the synthesis of amylose in potato [204]. The silencing construct contains an antisense *GBSS* gene composed of only potato sequences and is controlled by the potato *GBSS* promoter. However, the terminator is the one of the *nopaline synthase* gene (*nos*) from *A. tumefaciens*; thus, this crop could not be considered as completely intragenic. Nevertheless, this potato was released to the field in the EU in 2007 (B/NL/07/04) with the potato *GBSS* terminator, i.e., a fully intragenic potato plant. Another intragenic potato was engineered to reduce the acrylamide content in processed potatoes (without yield penalty or affecting the tuber shape) by silencing one *asparagine synthase* gene (*StAs1*) [205]. The development of other intragenic potatoes was achieved by overexpressing the *lycopene b-ciclase* (*StLYCb*) gene controlled by the potato *GBSS* promoter, which incited β-carotene accumulation in potato tubers [27]. In the case of tomato, carotenoid and flavonoid contents were enhanced simultaneously through the suppression of the *DE-ETIOLATED1* (*DET1*) gene by using RNAi technology and fruit-specific promoters [28]. A gluten-free wheat has also been obtained using this technology by silencing a γ-gliadin gene [206]. The iron content in wheat flour has been increased by more than 2-fold following the expression of a *vacuolar iron transporter* gene (*TaVIT2*) under the control of a wheat endosperm-specific promoter [12]. Finally, Dupont-Pioneer and Monsanto have developed two high oleic soybean oils, Plenish^®^ and Vistive^®^ Gold, respectively, which are currently available in the USA market.

## 5. Regulation of Plant Breeding Methods

The current regulatory framework could present an obstacle when the above-described techniques are used in crop biofortification, except for conventional breeding, which is not subject to any specific law. However, this is not the case for modern biotechnology techniques. Genetically modified (GM) crops have been demonstrated to be safe countless times, as supported by more than 100 Nobel laureates [231]. In addition, thousands of risk assessments conducted by independent federal regulatory agencies on GM crops have found that there is not different risks between GM and non-GM crops [232]. Nevertheless, there is a widespread lack of acceptance associated with the artificial combination of foreign genetic elements and the use of antibiotic or herbicide resistance selectable markers. All this has triggered alerts about potential health and environmental risks in case gene flow from GM to other non-GM crops [233]. Furthermore, the legislation continues to be strict and differs largely in each country.

In 2019, genetically engineered crops were cultivated in 29 countries, covering a total of 190 million hectares worldwide [232]. North and South America are the biggest producers, followed by Asia, where the law is more flexible. In fact, out of these 190 million hectares of biotech crops cultivation, 174 (90% of the total area) are located in only five countries: USA, Brazil, Argentina, Canada, and India (sorted in descending order) [232]. In the case of the European Union (EU), GMO regulation is one of the most severe, since it assumes that GM crops are intrinsically different (potentially dangerous) [234]. Thus, most countries have used the opt-out clause in relation to the GM crop cultivation and only six countries allow it, having permitted only the cultivation of a GM crop, Bt maize. This led to a decline in research and development (RD) investment in Europa from one-third of the global expenses in agriculture in the mid-1990s to less than 10% by 2013 [235]. Nevertheless, it is worthy to remark that, in England, the rules have been recently relaxed as a consequence of Brexit. Field trials of gene-edited crops with research purposes will be allowed without the current impediments and “red tape”, being only necessary to notify it to the Department for Environment, Food, and Rural Affairs (DEFRA) (https://www.gov.uk/government/news, accessed on 3 March 2022). In addition, these measures are likely to be extended to the rest of UK and a redefinition of the law about genetic modification is also expected. However, until then, gene-edited plants will still be considered GMOs and their commercial cultivation will have to be authorized under the actual law. In many African countries, there is either not any regulatory framework, or it is very restrictive, in spite of being regarded as the part of the world with the largest potential to benefit from the adoption of GM crops due to the high rates of hunger and malnutrition. Notwithstanding, the number of countries embracing GM crops in this continent has been doubled from three in 2018 to six in 2019 [232].

Despite the huge number of developed crops with enhanced traits through genetic engineering, only four different biotech crops cover more than 95% of the cultivated area (soybean, maize, cotton, and canola) and, in most cases, the modified traits are related to herbicide tolerance and insect resistance [232]. Therefore, additional efforts are needed to approve GM crops with enhanced nutritional value in order to contribute to the end of world hunger. Nowadays, transgenesis, cisgenesis, and intragenesis are subject to the same regulation in the vast majority of countries. However, cisgenic and intragenic crops are generally more accepted by the general public and are expected to be regulated less severely in the coming years in some countries [236]. In fact, in Canada, the regulation system is based on the final product rather than on the process to obtain it, which has relaxed the control of these kinds of crops in comparison with the transgenic ones [237]. In Australia, cisgenic plants are not considered GMOs, as stated in Gene Technology Regulations, whereby organisms that are not GMO include “a mutant organism in which the mutational event did not involve the introduction of any foreign nucleic acid” [238]. Other countries are also evaluating cisgenic and intragenic crop regulation. For example, in 2012, the European Food Safety Authority (EFSA) proposed a less precautionary approach to regulate cisgenesis, as it is supposed to entail similar hazards to conventional breeding as introduced by unmodified genes [239]. In the case of intragenesis, the EFSA affirmed that hazards are less predictable due to the recombination of different genetic elements, despite belonging to the same gene pool [240]. However, crops developed by RNAi technology, considered an intragenic approach, have recently received a positive opinion from this organization after determination of risk assessments [240]. In USA, the Environmental Protection Agency (EPA) is also discussing a less strict regulatory framework for cisgenesis and intragenesis approaches, especially when enhanced traits are related to pest resistance [241]. Furthermore, a lot of studies have confirmed a higher consumer and farmer acceptance of cisgenic and intragenic crops than transgenic ones because they are considered to be more natural [227,228,229]. This, together with the favorable opinions about cisgenesis and intragenesis from public organizations, should pave the way to less stringent regulations for these types of crops. Furthermore, a recent worldwide study has shown that consumers are willing to pay up to 23.9% more for GM-biofortified crops [242].

## 6. Future Perspectives

The Sustainable Development Goal 2 of the United Nations (UN) consists of ending all forms of hunger, including hidden hunger, before 2030. Nevertheless, projections show that unless serious actions are taken to accelerate the process, hunger will not be eradicated by that year. In fact, current progress is stalled or worsening [1]. Biofortification could substantially help to achieve that objective, as there are cost-effective strategies available. The technologies to explore genomic (i.e., SNP genotyping) and metabolic diversity are evolving astonishingly fast and becoming more and more high-throughput and, at least in the first case, affordable. Similarly, the approaches to identify and introduce the genomic regions responsible for the crop biortification, in this case, are becoming more accurate. However, all of them present some limitations, as we have discussed before. In the case of conventional breeding, the lack of genetic variability and the investment of time, although alleviated by the use of genomic tools (Table 4), to some extent, make it an insufficient strategy to reach the expected food demands [243]. Modern biotechnological techniques allow us to overcome those hurdles, though they are hampered by regulatory barriers, either non-existing specific laws or especially strict ones, as described in the previous section. Technology is progressing faster than the regulations and this gap is holding us back, for instance, to achieve the UN Sustainable Development Goals.

In parallel, an effort to illuminate the safety of genetically engineered crops in a clear and understandable manner is essential in order to increase their acceptance among the general public and political organizations. It would be also interesting to improve research and development of biofortified crops in developing countries, where malnutrition is a real burden.

## 7. Conclusions

Considering the expected increase in population in the next years, the challenge is not only to produce enough quantity of food to feed the global population, but also to ensure that food is nutritionally rich to ensure balanced diets. It is well established that biofortification is a cost-effective strategy and a promising approach to fight against global hunger, especially in developing countries. Currently, a large number of biofortified crops have been developed and even released, mainly those obtained through conventional breeding, but also some of them through modern biotechnological techniques. Nevertheless, GMO rejection implies an obstacle and it is frequently based on political preferences in spite of scientific evidences that support the safety of GM-biofortified crops. Here, it is necessary to set aside political and populist views not built on scientific results in order to guarantee food security, a global priority matter. Thus, the likely approval of cisgeneic and intragenic crops, and the less likely but also possible approval of transgenic ones, combined with conventional breeding and genome editing technologies, would place us closer and faster to the zero-hunger goal.

## Figures and Tables

**Table 1:** List of the main public SNP databases in food crops. The type of information available ranges from the marker description to the genotype and map and/or genome location.

Database Name	Url	Crop ^‡ ^
CerealsDB	https://www.cerealsdb.uk.net/cerealgenomics/CerealsDB/indexNEW.php, accessed on 17 January 2022	Bread wheat (*Triticum eastivum* L.)
Chickpea SNP-InDel Database (CicArVarDB)	https://cegresources.icrisat.org/cicarvardb, accessed on the 17 of January 2022	Chickpea (*Cicer arietinum* L.)
CropSNPdb	http://snpdb.appliedbioinformatics.com.au/, accessed on 17 January 2022	Bread wheat (*T. eastivum* L.) Cabbage (*Brassica rapa* L.) Cauliflower (*Brassica oleracea* L.) Indian mustard (*Brassica juncea* L.) Oilseed rape (*Brassica napus* L.)
Cucurbit Genomics Database (CuGeDG)	http://cucurbitgenomics.org/, accessed on 17 January 2022	Cucumber (*Cucumis sativus* L.) Melon (*Cucumis melo* L.) Pumpkin (*Cucurbita* spp.) Watermelon (*Citrullus lanatus* Thumb.)
Genome Database for Rosaceae (GDR)	https://www.rosaceae.org, accessed on 17 January 2022	Apple tree (*Malus* spp.) Blackberry (*Rubus* spp.) Peach tree (*Prunus* spp.) Pear tree (*Pyrus* spp.) Strawberry (*Fragaria* spp.)
Gramene	https://www.gramene.org, accessed on 17 January 2022	African rice (*Oryza galberrina* Steud) Asian rice (*Oryza sativa* L.) Barley (*Hordeum vulgare* L.) Foxtail millet (*Setaria italica* (L.) Beauv.) Maize (*Zea mays* L.) Sorghum (*Sorghum bicolor* (L.) Moench) Wheat (*Triticum* spp.)
Kazusa Tomato Genomics Database (KaTomicsDB)	https://www.kazusa.or.jp/tomato/, accessed on 17 January 2022	Tomato (*Solanum lycopersicum* L.)
Lettuce Genome Database (LettuceGDB)	https://www.lettucegdb.com, accessed on 17 January 2022	Lettuce (*Lactuca sativa* L.)
Maize Genetics and Genomics Database (MaizeGDB)	https://www.maizegdb.org/, accessed on 17 January 2022	Maize (*Z. mays* L.)
Maize SNP-DNA Fingerprint Database	http://doi.org/10.3390/agriculture11070597 (Tables S1 and S2; [127]), accessed on 18 January 2022	Maize (*Z. mays* L.)
Q-TARO (QTL Annotation Rice Online) database	http://qtaro.abr.affrc.go.jp/index.html, accessed on 18 January 2022	Asian rice (*O. sativa* L.)
SNP genotype database for avocado	https://doi.org/10.1007/s11295-019-1374-1 (Table S2; [128]), accessed on 18 January 2022	Avocado (*Persea americana* Mill.)
Sol Genomics Network	https://solgenomics.net, accessed on 18 January 2022	Tomato (*S. lycopersicum* L.)
SorGSD	https://ngdc.cncb.ac.cn/sorgsd, accessed on 18 January 2022	Sorghum (*S. bicolor* (L.) Moench)
SpinachBase	http://www.spinachbase.org, accessed on 19 January 2022	Spinach (*Spinacia oleracea* L.)
Rice SNP-Seek Database	https://snp-seek.irri.org, accessed on 19 January 2022	Asian rice (*O. sativa* L.)
The IPK Crop EST Database (CR-EST)	http://pgrc.ipk-gatersleben.de/cr-est, accessed on 19 January 2022	Barley (*H. vulgare* L.) Bread wheat (*T. eastivum* L.) Pea (*Pisum sativum* L.) Potato (*Solanum tuberosum* L.)
The Tomato Integrated Database (Tomatonics)	http://plantomics.mind.meiji.ac.jp/tomatomics, accessed on 19 January 2022	Tomato (*S. lycopersicum* L.)
TropGENE-DB	http://tropgenedb.cirad.fr/tropgene/JSP/index.jsp, accessed on 19 January 2022	Asian rice (*O. sativa* L.) Banana (*Musa acuminata* Juss.) Bread fruit (*Artocarpus altilis* (Parkinson) Fosberg) Cassava (*Manihot esculenta* Crantz) Clemetine (*Citrus clementina* L.) Cocoa (*Theobroma cacao* L.) Coconut (*Cocos nucifera* L.) Coffee (*Coffea canephora* L.) Cupuassu (*Theobroma grandiflorum* Schum.) Oil palm (*Elaeis guineensis* Jacq.) Pummelo (*Citrus grandis* (L.) Osbeck) Sorghum (*S. bicolor* L. Moench) Sugarcane (*Saccharum officinarus* L.) Sweet orange (*Citrus sinensis* Osbeck)
Vitis International Variety Catalogue (VIVC)	https://www.vivc.de/index.php?r=site%2Findex, accessed on 19 January 2022	Grapevine (*Vitis* spp.)

**^‡^** Even if there are more species in some databases, they were not included if there is no SNP information available or they are not food crops.

**Table 2:** Metabolomic genome-wide association studies (mGWAS). Only groups of compounds that play an important role in human nutrition and/or health status are shown.

Crop	Species	Analytical Technique ^‡^	Metabolite	Reference
Apple tree	*Malus* × *domestica* Borkh.	UHPLC–ESI-QTOF-MS, NMR	Flavonoids, polyphenols, sugars, terpenoids	[141]
Barley	*H. vulgare*	HPLC-FL, HPLC-MS, IC-MS/MS	Amino acids, glutathione, organic acids, starch, sugars, vitamin E (tocopherol)	[142]
		HPAEC-PAD, HPLC-ELSD, HPLC-MALDITOF-MS	Sugars	[143]
		HPLC-Fluorescence detection	Carotenoids (i.e., tocopherols and tocotrienols: vitamin E)	[144]
Barley Bread wheat Maize Potato Rice Sweet orange tree	*H. vulgare* *T. aestivum* *Z.mays* *S. tuberosum* *O.sativa* *Citrus xsinensis* (L.) Osbeck	GC-TOF-MS	Flavonoids	[145]
Blueberry	*Vaccinium* spp.	GC-MS	Fatty acids, phenylpropanoids, terpenoids	[146]
Bread wheat	*T.* *aestivum*	GC-MS	Amino acids, organic acid ^‡‡^ sugars	[147]
Foxtail millet	*S.* *italica*	HPLC-ESI-QTRAP-MS/MS	Alkaloids, amino acids, fatty acids, organic acids, phenolamides, polyphenols (i.e., flavonoids, anthocyanins...), sugars, vitamins	[148]
Lettuce	*L.* *sativa*	GC-TOF-MS	Alkaloids, amino acids, organic acids, polyamines, polyphenols, sugars, vitamins, etc.	[149]
Loquat	*Eriobotryajaponica* (Thunb.) Lindl.	UPLC-ESI-MS/MS	Alkaloids, flavonoids, phenolic acids, polysaccharides, terpenoids	[150]
Maize	*Z.* *mays*	LC-MS/MS	Fatty acids	[151]
		LC-ESI-(QTRAP or QqTOF)-MS/MS	Amino acids, fatty acids, flavonoids	[152]
		GC-MS	Amino acids, organic acids, phenylpropanoids	[153]
		HPLC-Fluorescence detection	Tocochromanols (tocopherols and tocotrienols)	[154]
		HPLC-PDA	Carotenoids	[155]
		UPLC-HRMS	Amino acids, fatty acids, flavonoids, benzoxazinoids, terpenoids	[156]
		HPLC, UPLC	Carotenoids	[157]
		CEC	Amino acids	[158]
		LC-ESI-QqTOF-MS/MS	Flavonoids	[159]
		HPLC	Carotenoids	[160]
		UPLC-PDA	Tocopherol (part of vitamin E)	[161]
		GC-TOF-MS	Amino acids, (poly)amines, organic acids, sugars, vitamin E (tocopherol)	[162]
		HPLC-PDA, HPLC-fluorescence detection	Carotenoids, phenolics, tocopherol (a form of vitamin E)	[163]
		HPLC-fluorescence detection	Carotenoids (i.e., tocopherols and tocotrienols: vitamin E)	[164]
		HPLC-PDA	Carotenoids (α-carotene, β-carotene, β-cryptoxanthin, lutein, phytofluene, zeaxanthin, zeinoxanthin)	[165]
		HPLC-UV/Vis	Anthocyanins	[166]
Potato	*S.* *tuberosum*	UPLC-Q-TOF-MS	Alkaloids, amino acids	[167]
Rice	*O.* *sativa*	LC-ESI-Q TRAP-MS/MS	Phenolamides	[168]
		GC-TOF-MS	Amino acids, flavonoids, organic acids	[169]
		LC-ESI-MS/MS	Amino acids, fatty acids, flavonoids	[170]
		HPLC-ESI-QTOF/MS	Amino acids, flavonoids, phenolamines, terpenoids	[171]
		HPLC-ESI-(QTRAP or QqTOF)-MS	Amino acids, flavonoids, phenolamines, terpenoids	[70]
		LC-ESI-Q TRAP-MS/MS	Flavonoids	[172]
Soybean	*Glycinemax* L.	GC	Fatty acids	[173]
		HPLC-DAD	Isoflavones	[174]
		HPLC-MS	Aminoacids, isoflavones, lipids, organic acids	[175]
Tea	*Camelliasinensis* L.	HPLC	Theanine, caffeine, catechins	[176]
		HPLC-PDA	Amino acids, caffeine, catechins	[177]
Tomato	*S.* *lycopersicum*	GC-MS	Organic acids, sugars	[178]
		GC-MS	Amino acids, organic acid ^‡‡^, sugars	[179]
		HPLC-MS/MS	Alkaloids ^‡‡‡^	[180]
		GC-MS	Fatty acids, lipids, carotenoids (i.e., tocopherols and tocotrienols: vitamin E)	[181]
Wheat	*T.* *aestivum*	HPLC-ESI-QTRAP-MS/MS	Amino acids, (poly)amines, flavonoids, organic acids, sugars, vitamins, etc.	[182]

**^‡^** CEC: cation exchange chromatography; ELSD: evaporative light scattering detection; GC: gas chromatography; GC-MS: GC mass spectrometry; GC-TOF-MS: GC time-of-flight mass spectrometry; HPAEC-PAD: high-pH anion-exchange chromatography with pulsed amperometric detection; HPLC: high-performance liquid chromatography; HPLC-ESI-(QTRAP or QqTOF)-MS: HPLC-ESI-quadrupole TRAP or TOF tandem mass spectrometry; HPLC-MALDITOF-MS: HPLC matrix-assisted laser desorption–ionization time-of-flight mass spectrometry; IC-MS/MS: ion chromatography tandem mass spectrometry; LC-ESI-MS/MS: liquid chromatography–electrospray ionization tandem mass spectrometry; LC-Q-TOF-MS: liquid chromatography quadrupole TOF mass spectrometry; NMR: nuclear magnetic resonance; UPLC-ESI-MS/MS: ultra-high-performance liquid chromatography ESI tandem mass spectrometry; UPLC-HRMS: UPLC high-resolution mass spectrometry. **^‡‡^** Oxalic acid (anti-nutrient). **^‡‡‡^** Steroidal glycoalkaloids (SGAs): most of them are considered anti-nutrients.

**Table 3:** Biofortified crops through different techniques.

Technique	Crop	Method	Biofortified Trait	Reference
Conventional breeding	Rice	Backcrosses between a high-yielding cultivar and the IR68144 line	A 2.54-fold increase in iron and 1.54-fold increase in zinc	[4]
	Maize	Backcrosses involving diverse exotic donor lines	Lines with high provitamin A content by accumulating mainly high β-carotene and lines with high provitamin A by promoting accumulation of high levels of both carotenes and xanthophylls	[13]
		Marker-assisted introgression of *lpa1-1* and *lpa2-1* alleles in elite lines of provitamin A-enriched quality protein maize (QPM)	A reduction in phytic acid content and improvement in the mineral bioavailability in lines of QPM rich in provitamin A	[197]
		Introgression of *VTE4* (γ-tocopherol methyl transferase) allele into four provitamin-A rich QPM elite inbreds using marker-assisted backcross breeding	An increase in α-tocopherol to 15.2 ppm over 8.0 ppm in the original inbreds	[14]
	Wheat	Marker-assisted introgression of group 4 and 7 chromosomes of the wild ancestor *Aegilops peregrina* in a commercial variety of wheat	Higher content in iron and zinc in wheat grains	[5]
		Backcrosses between low-yielding exotic donor lines and commercial varieties	Black, purple, and blue lines with high content in anthocyanins	[38]
	Cassava	Rapid cycling recurrent selection	Significant gains for total carotenoid content and total β-carotene	[15]
	Potato	‘Atlantic’ and 17 4x-2x hybrids between *S. tuberosum* and diploid hybrids of *Solanum phureja-Solanum stenotomum*	Higher contents of copper, iron, manganese, and zinc	[6]
	Tomato	Backcrosses between landraces of tomato	Hybrid with increased concentration of polyphenols and high antioxidant activity in pink ripeness stage	[39]
	Bean	Backcrosses between low and high mineral genotypes using a QTL mapping approach	Increased iron and zinc content	[7]
	Chickpea	Crosses between different cultivars	Higher content of carotenoids	[16]
Transgenesis	Rice	Endosperm-specific overexpression of *Arabidopsis thaliana GTP cyclohydrolase I* (*GTPCHI*) and *aminodeoxychorismate synthase* (*ADCS*) genes	An enhancement of 100 times in folate	[198]
		Overexpression of *phytoene synthase*	Higher content in β-carotene	[17]
		Expression of four synthetic genes: *sZmPSY1, sPaCrtI, sCrBKT*, and *sHpBHY* (for phytoene synthase, phytoene desaturase, β-carotene ketolase, and β-carotene hydroxylase, respectively)	Synthesis de novo of the carotenoid astaxanthin	[40]
		Coexpression of an *Arabidopsis* nicotianamine synthase (*AtNAS1*), bean ferritin (*PvFerritin*), bacterial carotene desaturase (*CRTI*), and maize phytoene synthase (*ZmPSY*)	Simultaneous increase in iron, zinc, and β-carotene content in the rice endosperm	[29]
		Constitutive overexpression of the rice *GDP-L-galactose phosphorylase* (35S-*OsGGP*) gene	Increase in ascorbate concentrations in germinated brown rice	[18]
		Expression bacterial *aspartate kinase* (*AK*) and *dihydrodipicolinate synthase* (*DHPS*), downregulation of rice *lysine ketoglutarate reductase/saccharopine dehydrogenase (LKR/SD)* and selection of marker-free transgenic lines	Up to 25-fold increase in free lysine levels	[36]
		Expression of an AmA1 gene from *Amaranthus hypochondriacus*	A significant increase in the content of several EAAs, including lysine, threonine, and valine, as well as a 1.06~12.87% increase in the total protein content	[37]
	Maize	Overexpression of the bacterial genes *crtB* (for phytoene synthase) and *crtI* (for the four desaturation steps of the carotenoid pathway) under the control of a endosperm-specific promoter	An increase in total carotenoids of up to 34-fold with a preferential accumulation of β-carotene in the maize endosperm	[19]
		Endosperm-specific overexpression of soybean ferritin	A 2-fold improvement in seed iron bioavailability	[8]
		Coexpression of *Gm8gGCHI* and *GmADCS* genes driven by endosperm-specific promoters	A 4.2-fold increase in folate (vitamin B9) level in transgenic maize grains	[20]
		Insertion of the lysine-rich *sb401* gene	Significantly higher levels of lysine total protein in maize seeds	[35]
	Wheat	Constitutive expression of the rice *nicotianamine synthase 2* (*OsNAS2*) gene	Higher concentrations of grain iron and zinc, and enhanced localization of iron and zinc in endosperm and crease tissues, respectively	[9]
	Cassava	Coexpression of *ferritin* (*FER1*) and mutated *Iron transporter* (*IRT1*) from *A. thaliana*	Accumulation of iron levels 7–18 times higher and zinc levels 3–10 times higher	[10]
	Potato	Overexpression of *AtGTPCHI, AtADCS, OsHPPK/DHPS* and *AtFPGS* genes	A 2-fold increase in folate content in mature tubers and stable accumulation of folates for up to 9 months of storage	[21]
		Simultaneous expression of *Wrinkled 1* (*WRI1*), *Diacylglycerol acyltransferase 1* (*DGAT1*) and *Oleosin* under the transcriptional control of tuber-specific (patatin) and constitutive (CaMV-35S) promoters.	Over a 100-fold increase in triacylglycerol accumulation to levels up to 3.3% of tuber dry weigh	[33]
	Sweet Potato	Expression of a barley *NA synthase 1* (*HvNAS1*) gene	A 3- and 2.9-fold increase in the concentrations of iron and zinc, respectively	[11]
	Tomato	Cross between *GTPCHI* and *ADCS* overexpressing plants	A 25-fold more in folate (Vitamin B9) level in fruits	[199]
		Overexpression of an *A. thaliana* *Orange* (*AtOR*) gene	An increase in total carotenoids in fruits	[22]
		Overexpression of *GDP-l-galactose phosphorylase* (*GGP*) gene from *Actinidia chinensis* under the control of the 35S promoter	A 3- to 6-fold higher content in ascorbic acid in fruits	[200]
		Fruit-specific expression of the transcription factor *AtMYB12*	Increased content of different phenylpropanoids	[23]
	Strawberry	Overexpression of a *GDP-l-galactose phosphorylase* (*GGP*) gene from *Actinidia chinensis* under the control of the 35S promoter	A 2-fold higher content in ascorbic acid in fruits	[200]
	Banana	Expression of a *Fe’i banana-derived phytoene synthase* (*MtPsy2a*) gene under the maize polyubiquitin promoter	Enhanced β-carotene content in fruit	[24]
	Soybean	Overexpression of the bacterial genes *crtB* (for phytoene synthase) and *crtW* and *bkt1* (ketolase genes) under the control of seed-specific promoters	Enhanced accumulation of ketocarotenoids in seeds	[201]
		Overexpression of adenosine 5’-phosphosulfate sulfurylase 1	Higher amounts of sulfate, cysteine, and some sulfur-containing secondary metabolites in seeds	[34]
		Overexpression of a *GmDGAT2A* gene driven by a seed-specific promoter of *Gmole1*	Significantly increased linoleic acid content specifically and total oil content	[32]
	Bean	Seed-specific overexpression of a *GTP cyclohydrolase I* gene from *Arabidopsis* (*AtGchI*)	Increased folate levels in raw desiccated seeds by up to 3-fold	[25]
	Canola	Downregulation of lycopene ε-cyclase (*ε-CYC*)	Increased levels of β-carotene, zeaxanthin, violaxanthin, and lutein	[26]
	*Brassica* *carinata*	Expression of an *18-carbon ω3 desaturase* (*CpDesX*) gene from *Claviceps purpurea* and a *20-carbon ω3 desaturase* (*Pir-ω3*) gene from *Pythium irregulare*	Up to 25% increase in eicosapentaenoic acid	[31]
	Linseed	Expression of a Δ6-desaturase from *Primula vialii*	Transgenic lines that accumulate the omega-3 fatty acid stearidonic acid	[30]
Cisgenesis	Barley	Expression of a barley *phytase* gene (*HvPAPhy_a*)	Decrease in phytate concentration, which then increases phosphate bioavailability	[202]
	Potato	Suppression of a *starch phosphorylase* L. gene through dsRNAi technology	Decrease in starch degradation what reduces the accumulation of reducing (glucose, fructose) and non-reducing (sucrose) sugars in tubers stored at 4 °C	[203]
	Apple	Expression of *MdMYB10* transcription factor	Red-fleshed ‘Gala’ apples rich in anthocyanins	[41]
Intragenesis	Potato	Silencing of a *granule-bound starch synthase* (*GBSS*) gene	An increase in amylopectin content	[204]
		Silencing of an *asparagine synthase gene* (*StAs1*)	Reduced free asparagine concentration by up to 80% and consequent decrease in acrylamide content in processed potato	[205]
		Overexpression of a *lycopene b-cyclase* (*StLYCb*) gene under the GBSS promoter	An increase in β-carotene accumulation in potato tubers	[27]
	Tomato	Suppression of a *DE-ETIOLATED1* (*DET1*) gene through RNAi technology	Enhanced carotenoid and flavonoid content	[28]
	Wheat	Suppression of a *γ-gliadin* gene by using RNAi technology	Gluten-free wheat	[206]
		Overexpression of a *vacuolar Iron transporter* (*TaVIT2*) under the control of a wheat endosperm-specific promoter	An increase in more than 2-fold of iron in white flour fractions	[12]
	Soybean	RNAi technology	Plenish^®^ high oleic	Dupont-Pioneer (Johnston, IA, USA)
			Vistive^®^ Gold low saturated high oleic	Monsanto (St. Louis, MO, USA)

**Table 4:** Comparison of the main characteristics of conventional breeding, transgenesis, cisgenesis, and intragenesis.

Characteristic	Conventional Breeding	Transgenesis	Cisgenesis	Intragenesis
Variability source	Sexually compatible group	Any organism	Sexually compatible group	Sexually compatible group
Method	Crosses and selection	Recombinant DNA	By *Agrobacterium*	By *Agrobacterium* (recombinant DNA)
Introducing DNA	Natural	Natural and/or artificial	Natural	Natural and/or artificial
Gene pool	Unaltered	Altered	Unaltered	Altered
Borders	-	T-DNA	T-DNA (to be eliminated)	T-DNA or P-DNA
Linkage drag	Yes	No	No	No
Expression modulation	No	Yes	Yes	Yes
Time	High	Medium	Medium	Medium

## Data Availability

Not applicable.

## Supplementary Materials

The following are available online at https://www.mdpi.com/article/10.3390/ijms23063086/s1, Supplementary File S1: Bibliographic search criteria to elaborate the present review.
