# Supplementary material for: Applications of Genomic Tools in Plant Breeding: Crop Biofortification
Source: Int J Mol Sci. 2022 Mar 13;23(6):3086. doi: 10.3390/ijms23063086 (PMC8950180; doi:10.3390/ijms23063086)
Supplement: Supplementary file 1 [file ijms-23-03086-s001.zip › ijms-1600584-supplementary.pdf]

Bibliographic search criteria to elaborate the present review.

This review was performed by a search of international literature in the databases PubMed, Web of Science and Scopus, with the following search terms in several fields within the different sections:

1. Introduction: (“biofortification”) AND (“crop”); (“metabolomic”) AND (“crop”)

2. Exploring biodiversity: searching for outstanding material: (“SNP genotyping”) AND (“crop”);

3. Association between the traits of interest and the genomic regions: fishing for genes: (“GWAS”) AND (“metabolomic”) OR (“metabolic GWAS”) OR (“mGWAS”)

4. Introducing allelic variants to biofortify crops: (“breeding” OR transgen* OR cisgen* OR intragen*) AND (“biofortification”)); (cisgen* AND intragen*)

5. Regulation of plant breeding methods: (“GMO” OR “transgenic”) AND (“legislation”)); ((cisgen* OR intragen*) AND (“legislation”))

The retrieved list of references was manually screened, on one hand, to be filtered and, on the other hand, to look for additional publications relevant to this review. No limitations of date, language, or study design were established. In addition, data from international organizations like FAO, UNICEF, EFSA, UK Government etc., were also consulted. The searches were conducted during 2021 and beginning of 2022.
